# Blood pressure variability combined with coagulation function in early prediction and outcome assessment of germinal matrix-intraventricular hemorrhage in preterm infants with gestational age ≤32 weeks

**DOI:** 10.1371/journal.pone.0328904

**Published:** 2025-07-24

**Authors:** Lijun Jiang, Qian Yu, Hui Li, Fudong Wang, Feng Liu, Zhenxing Xu

**Affiliations:** 1 Department of Neonatology, Affiliated Hospital of Yangzhou University, Yangzhou, Jiangsu Province, China; 2 Department of Interventional Medicine, Yangzhou Hongquan Hospital, Yangzhou, Jiangsu Province, China; Nationwide Children's Hospital, UNITED STATES OF AMERICA

## Abstract

**Objective:**

To determine the association between blood pressure variability (BPV), coagulation indexes, and germinal matrix-intraventricular hemorrhage (GMH-IVH) in preterm infants with gestational age ≤ 32 weeks. In addition, we aimed to determine whether the combination can predict the occurrence and outcome of GMH-IVH.

**Methods:**

This retrospective study included 106 preterm infants. According to the presence of GMH-IVH, the preterm infants were divided into GMH-IVH (51 patients) and no GMH-IVH (55 patients) groups. Furthermore, according to the short-term prognoses, the GMH-IVH group was subdivided into good outcome (30 patients) and poor outcome (21 patients) groups. Coagulation function and BPV indexes were collected at admission. Univariate analysis, logistic regression model, and receiver operating characteristic curve were used to analyze the relationship between indexes and the occurrence and outcome of GMH-IVH in preterm infants.

**Results:**

Univariate analysis showed that the difference between maximum and minimum (Max-Min); standard deviation (SD); coefficient of variation (CV) of BPV, prothrombin time (PT), international normalized ratio (INR), activated partial thromboplastin time (APTT), and proportion of premature rupture of membranes (PROM) were higher in the GMH-IVH group than the no GMH-IVH group (*P < 0.05*). Logistic regression analysis showed that INR and DBP SD were directly correlated with GMH-IVH, and the joint curve had the largest area under the curve (AUC) (82.4% sensitivity and 79.7% specificity). BPV SD, BPV CV, APTT, and INR were higher in the poor outcome group than in the good outcome group (*P < 0.05*). Logistic regression analysis showed that INR and DBP SD were directly correlated with poor outcomes in preterm infants with GMH-IVH. The joint curve had the largest AUC (sensitivity 76.2% and specificity 90.0%).

**Conclusion:**

Increased INR and DBP SD are directly associated factors for the developement and poor short-term outcome of GMH-IVH, and combined monitoring of INR and DBP SD has certain reference value for the early identification and prognosis evaluation of GMH-IVH in preterm infants with gestational age ≤ 32 weeks.

## Introduction

In recent years, with advancements in neonatal intensive care technology, the survival rate of premature infants has been steadily increasing. However, the incidence of GMH-IVH in preterm infants with GA of ≤32 weeks has not shown a significant decrease. GMH-IVH can lead to adverse neurological outcomes and even mortality [1]. Given that the clinical manifestations of GMH-IVH in preterm infants are often subtle, this condition is prone to being overlooked during diagnosis. Therefore, early prediction and comprehensive management strategies to prevent the occurrence of GMH-IVH in preterm infants are crucial for improving their prognosis.

The pathogenesis of GMH-IVH is complex and may result from multiple factors. Among these, the dysregulation of cerebral blood flow (CBF) and the increased risk of bleeding due to immature coagulation function are critical factors contributing to GMH-IVH in preterm infants [2,3]. Currently, accurately assessing CBF in clinical practice remains challenging. Blood pressure (BP) fluctuations can indirectly indicate variations in CBF; however, BP is influenced and modulated by numerous factors and thus cannot reliably reflect changes in CBF. BPV refers to the variation in BP caused by various pathological and physiological factors. Compared with BP measurements, BPV provides a more accurate reflection of the body’s hemodynamic state [4]. Studies have demonstrated that BPV has independent predictive value for organ damage, cardiovascular and cerebrovascular events, as well as mortality in adults [5]. However, its role in early prediction and prognosis evaluation of brain injury in preterm infants has not been extensively reported.

We hypothesize that BPV can serve as a reflection of the cerebral hemodynamic status in preterm infants to some extent, and coagulation index analysis may indicate the potential risk of bleeding in this population. The integration of BPV with coagulation index analysis could enhance early prediction and prognosis evaluation of GMH-IVH. This study aims to explore the relationship between coagulation function, BPV, and GMH-IVH in preterm infants, as well as assess whether the combination of these two parameters can more effectively evaluate the development and short-term prognosis of GMH-IVH, thereby providing guidance for the prevention and management of GMH-IVH.

## Patients and methods

### Patient selection and group

The present study retrospectively analyzed the clinical data of premature infants admitted to the Neonatal Intensive Care Unit (NICU) at the Affiliated Hospital of Yangzhou University between June 2022 and June 2024. Based on the presence or absence of GMH-IVH, the patients were categorized into GMH-IVH and non-GMH-IVH groups. Furthermore, according to their short-term outcomes, the preterm infants in the GMH-IVH group were subdivided into good outcome and poor outcome subgroups. This study was approved by the hospital’s local ethics committee (No. 2022-YKL3-06-005), and written informed consent was obtained from the parents of the participants.

Inclusion criteria: (1) Admission within one hour after birth; (2) GA ≤ 32 weeks; (3) No administration of blood products, anticoagulants, or inotropic agents. Exclusion criteria: (1) Central nervous system infection; (2) Congenital brain developmental abnormalities; (3) Congenital coagulation disorders; (4) Thrombocytopenia; (5) Inherited metabolic disorders.

### General data collection

The clinical and demographic characteristics of all enrolled infants were systematically collected, including gender, GA, birth weight, mode of delivery, 5-minute Apgar score, prenatal hormone administration, premature rupture of membranes (PROM), requirement for mechanical ventilation, presence of patent ductus arteriosus (PDA), and so on.

### Transcranial ultrasound

Doppler ultrasound was utilized to screen for GMH-IVH on the 1st, 3rd, and 7th day after birth. GMH-IVH was graded according to the criteria proposed by Papile [6]. Transcranial sonography was conducted using the 3–11 MHz, C11-3S probe, and examinations were carried out by two senior neonatologists with at least 10-year experience in neonatal transcranial sonography.

### Coagulation function

Coagulation tests were conducted on postnatal day 1, with blood samples collected via a non-heparinized peripheral venous cannula or umbilical arterial or venous catheter. The coagulation parameters assessed included prothrombin time (PT), international normalized ratio (INR), activated partial thromboplastin time (APTT), fibrinogen (FIB), and D-dimer (DD), which were measured using the Sysmex CS-5100 automatic blood coagulation analyzer.

### BPV data collection

Systolic blood pressure (SBP) and diastolic blood pressure (DBP) were monitored every 3 hours during the first 3 days after birth. The average of two measurements obtained in a quiet state was recorded. To minimize errors associated with repeated measurements, a 5-minute interval was maintained between the two measurements. Furthermore, all operators underwent standardized training prior to the study implementation. BPV was assessed using the difference between the maximum and minimum values (Max-Min), standard deviation (SD), coefficient of variation (CV), and continuous variation (SV) as indicators [7,8].

### Outcome evaluation

Outcome assessment for preterms with GMH-IVH included both brain injury evaluation and neurodevelopmental assessments. Brain injury was identified using cranial magnetic resonance imaging (MRI) at term-equivalent postmenstrual age [9]. Neurodevelopmental status was evaluated via the Neonatal Behavioral Neurological Assessment (NBNA) at a corrected GA of 40 weeks [10]. Indicators of short-term adverse outcomes included reduced periventricular white matter volume and cystic degeneration, lateral ventricular dilatation, thinning of the corpus callosum, delayed myelination, and an NBNA score < 37 [9,10].

### Statistical analyses

Statistical analyses were performed using SPSS software (version 26.0, Inc., Chicago, IL, USA). Kolmogorov–Smirnov test was used to test the normality of the data. For normally distributed measurement data, mean±SD was used. An independent sample *t*-test or analysis of variance was used to compare between groups. Median (interquartile range) was used for non-normally distributed data. U test was used for comparison between groups. Count data were expressed as relative percentages (%), and the *χ*^*2*^ test was used for data comparison. The indexes with *P < 0.05* were entered into the multi-collinearity test, and then the variables were further entered into the logistic regression model and analyzed by the receiver operating characteristic (ROC) curve. The ROC curve was drawn to determine the indexes with the best predictive power for diagnosis and outcome.

## Results

### Comparison of general data

A total of 106 preterm infants with a GA of ≤32 weeks who met the inclusion criteria were enrolled in this study (S1 Fig). Among them, 55 were non-GMH-IVH preterm infants and 51 were GMH-IVH preterm infants. No significant differences were observed in general characteristics between the non-GMH-IVH group and the GMH-IVH group (*P > 0.05*), except for PROM (*P < 0.05*) (Table 1).

**Table 1:** Comparison of general, n(%)/mean±SD.

Characteristics	No GMH-IVH (n = 55)	GMH-IVH (n = 51)	*t/χ2*	*P*
Gestational age (w, $\overset{―}{x}$ ± s)	29.4 ± 2.0	28.5 ± 2.4	1.904	0.060
Birth weight (g, $\overset{―}{x}$ ± s)	1270.0 ± 247.4	1196.0 ± 319.8	1.338	0.084
Gender (male), n %	22(40.0)	21 (41.2)	0.047	0.829
Vaginal delivery, n %	23 (41.8)	29 (56.9)	2.397	0.122
Multiple birth, n %	4 (7.3)	8 (15.7)	1.866	0.172
Small for gestational age, n %	8 (14.5)	5 (9.8)	0.553	0.457
Apgar at 5′ ≤ 7, n %	7 (12.7)	14 (27.5)	3.611	0.057
Gestational hypertension, n %	13 (23.6)	6 (11.8)	2.535	0.111
Gestational diabetes mellitus,n %	5 (9.1)	6 (11.8)	0.203	0.652
Invasive ventilation, n %	5 (9.1)	9 (17.6)	1.690	0.194
Antenatal corticosteroids, n %	18 (32.7)	24 (47.1)	2.272	0.132
Premature rupture of membrane, n %	9 (16.4)	18 (35.3)	4.995	0.025
Patent ductus arteriosus, n %	23 (41.8)	30 (58.8)	3.061	0.080

GMH-IVH, greminal matrix-intraventricular hemorrhage; SD, standard deviation.

### Coagulation function and early prediction of GMH-IVH

Univariate analysis revealed that PT, INR, and APTT levels were significantly higher in the GMH-IVH group compared to the non-GMH-IVH group (*P < 0.05*) (Table 2).

**Table 2:** Comparison of coagulation function between the no GMH-IVH and the GMH-IVH, mean±SD/ median (IQR).

Characteristics	No GMH-IVH (n = 55)	GMH-IVH (n = 51)	*t/Z*	*P*
PT (sec)	15.1 ± 3.1	16.4 ± 3.3	−2.107	0.037
INR	1.35 ± 0.27	1.62 ± 0.30	−4.916	<0.001
APTT (sec)	57.20 ± 17.74	67.04 ± 22.08	−2.537	0.013
FIB (g/L)	1.54 ± 0.62	1.45 ± 0.61	0.761	0.448
DD (mg/L)	4.409 (1.734,4.283)	5.049 (1.987,6.311)	−0.595	0.553

GMH-IVH, greminal matrix-intraventricular hemorrhage; PT, prothrombin time; INR, international normalized ratio; APTT, activated partial thromboplastin time; FIB, fibrinogen; DD, D-Dimer; SD, standard deviation; IQR, interquartile range.

### BPV and early prediction of GMH-IVH

Univariate analysis revealed that the Max-Min, SD, and CV for SBP and DBP were significantly higher in the GMH-IVH group compared to the non-GMH-IVH group (P < 0.05) (Table 3).

**Table 3:** Comparison of BPV between the no GMH-IVH and the GMH-IVH, mean±SD.

Characteristics	No GMH-IVH (n = 55)	GMH-IVH (n = 51)	*t*	*P*
SBP Mean (mmHg)	57.02 ± 5.79	56.08 ± 6.31	0.800	0.426
SBP Max-min (mmHg)	21.62 ± 6.68	25.08 ± 6.88	−2.625	0.010
SBP SD	5.27 ± 1.49	6.12 ± 1.72	−2.739	0.007
SBP CV (%)	9.58 ± 2.96	11.18 ± 3.68	−2.468	0.015
SBP SV	7.91 ± 1.27	8.22 ± 1.46	−1.167	0.246
DBP Mean (mmHg)	29.11 ± 4.79	26.80 ± 5.31	2.349	0.021
DBP Max-min (mmHg)	21.65 ± 6.92	25.84 ± 5.91	−3.337	0.001
DBP SD	5.61 ± 1.62	6.64 ± 1.32	−3.558	0.001
DBP CV (%)	20.02 ± 7.74	25.72 ± 7.21	−3.914	<0.001
DBP SV	7.24 ± 1.19	7.68 ± 1.45	−1.705	0.090

GMH-IVH, greminal matrix-intraventricular hemorrhage; SBP, systolic blood pressure; DBP, diastolic blood pressure; Max-min, difference between maximum and minimum; SD, standard deviation; CV, coeffcient of variation; SV, successive variation.

### Logistic regression analysis of directly associated factors for GMH-IVH

Binary logistic regression analysis revealed that the INR and DBP SD were the most significant independent predictors associated with GMH-IVH, with an OR (95% CI) of 5.608 (2.858–8.587) and 1.455 (1.003–2.111), respectively (Table 4).

**Table 4:** Logistic regression analysis of directly correlated factors for GMH-IVH.

Variables	*β*	*SE*	*Wald χ* ^*2*^	*OR (95%CI)*	*P*
PROM	0.357	0.553	2.415	1.528(0.483-3.224)	0.519
PT	0.154	0.102	1.052	1.045(0.730-1.384)	0.244
INR	2.929	0.992	8.711	5.608(2.858-8.587)	0.003
APTT	0.021	0.016	0.107	1.028(0.972-1.603)	0.909
SBP Max-min	0.011	0.044	0.062	1.010(0.927-1.099)	0.826
SD	0.345	0.279	2.013	1.115(0.818-1.622)	0.417
CV	0.132	0.176	0.567	1.052(0.809-1.611)	0.451
DBP Mean	−0.031	0.50	0.377	0.970(0.876-1.165)	0.482
Max-min	0.002	0.044	0.003	1.109(0.922-1.295)	0.908
SD	0.690	0.465	2.852	1.455(1.003-2.111)	0.038
CV	0.305	0.191	1.195	1.093(0.800-1.962)	0.139

PROM, Premature rupture of membrane; PT, prothrombin time; INR, international normalized ratio; APTT, activated partial thromboplastin time; SBP, systolic blood pressure; DBP, diastolic blood pressure; Max-min, difference between maximum and minimum; SD, standard deviation; SE, Standard error; OR, Odds ratio; CI, Confdence interval.

### The predictive values of INR and DBP SD for GMH-IVH

**Fig 1:**
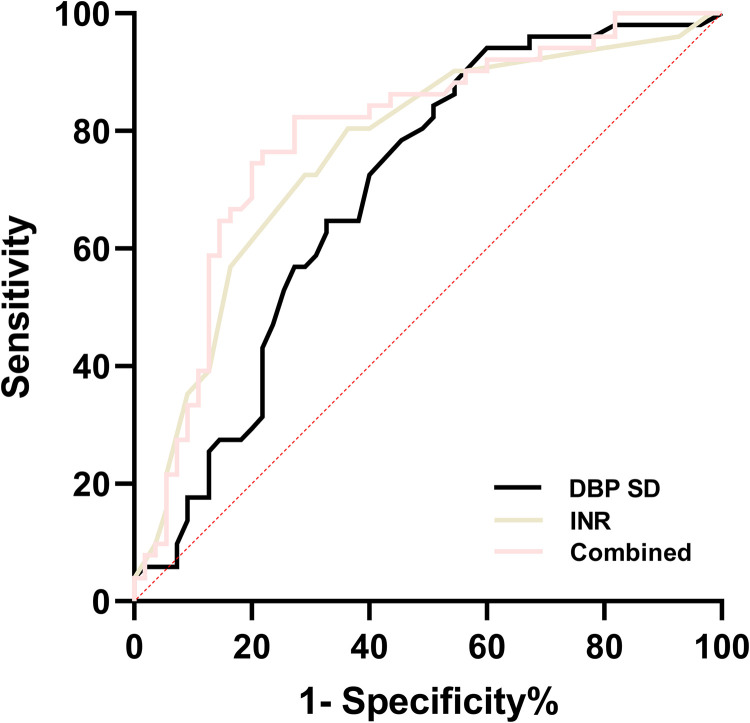
ROC curve of directly correlated factors for GMH-IVH. Combined = DBP SD + INR; DBP, diastolic blood pressure; SD, standard deviation; INR, international normalized ratio.

The ROC curve was constructed, and the area under the curve (AUC) for the combination of INR and DBP SD was determined to be the largest, with a value of 0.803. When the cut-off value was set at 0.41, the Youden index reached its maximum, with corresponding sensitivity and specificity values of 82.4% and 79.7%, respectively (Table 5 and Fig 1).

**Table 5:** The predictive values of INR, DBP SD, and their combination for GMH-IVH.

	AUC	95% *CI*	Cut-off	*P*	Sensitivity (%)	Specifcity (%)	Youden’s index
INR	0.764	0.671-0.856	1.565	<0.001	72.5	70.9	0.434
DBP SD	0.727	0.629-0.824	5.750	<0.001	78.4	68.5	0.469
Combined	0.803	0.724-0.881	0.405	<0.001	82.4	79.7	0.621

Combined = DBP SD + INR; DBP, diastolic blood pressure; SD, standard deviation; INR, international normalized ratio; AUC, area under the curve; CI, Confdence interval.

### Coagulation function and outcome evaluation of GMH-IVH

The INR and APTT were significantly higher in the poor outcome group compared to the good outcome group (*P < 0.05*) (Table 6).

**Table 6:** Comparison of coagulation function between the good and the poor outcome group, mean±SD/ median (IQR).

Characteristics	Good outcome (n = 30)	Poor outcome (n = 21)	*t/Z*	*P*
PT (sec)	16.1 ± 2.9	16.8 ± 3.7	−0.675	0.503
INR	1.53 ± 0.25	1.74 ± 0.34	−2.489	0.016
APTT (sec)	61.3 ± 19.8	75.2 ± 23.1	−2.298	0.026
FIB (g/L)	1.56 ± 0.61	1.28 ± 0.57	1.651	0.105
DD (mg/L)	4.823 (1.875,6.325)	5.372 (2.116,6.528)	−0.368	0.715

PT, prothrombin time; INR, international normalized ratio; APTT, activated partial thromboplastin time; FIB, fibrinogen; DD, D-Dimer; IQR, interquartile range.

### BPV and outcome evaluation of GMH-IVH

The SD and CV of SBP and DBP were higher in the poor outcome group than in the good outcome group (*P < 0.05*) (Table 7).

**Table 7:** Comparison of BPV between the good and the poor outcome group, mean±SD.

Characteristics	Good outcome (n = 30)	Poor outcome (n = 21)	*t/Z*	*P*
SBP Mean (mmHg)	56.63 ± 6.64	55.29 ± 5.88	0.747	0.459
SBP Max-min (mmHg)	24.80 ± 6.18	25.48 ± 7.93	−0.342	0.734
SBP SD	5.74 ± 1.73	6.75 ± 1.56	−2.139	0.037
SBP CV (%)	10.13 ± 3.61	12.33 ± 3.20	−2.243	0.029
SBP SV	8.08 ± 1.65	8.43 ± 1.14	−0.846	0.402
DBP Mean (mmHg)	27.97 ± 6.12	24.90 ± 3.55	2.059	0.045
DBP Max-min (mmHg)	24.87 ± 6.21	27.24 ± 5.29	−1.425	0.161
DBP SD	6.14 ± 1.22	7.38 ± 1.10	−3.710	0.001
DBP CV (%)	23.43 ± 6.75	29.21 ± 6.70	−3.022	0.004
DBP SV	7.37 ± 1.53	8.12 ± 1.25	−1.857	0.069

SBP, systolic blood pressure; DBP, diastolic blood pressure; Max-min, difference between maximum and minimum; SD, standard deviation; CV, coeffcient of variation; SV, successive variation.

### Logistic regression analysis of directly associated factors for outcome of GMH-IVH

Binary logistic regression analysis revealed that the INR and DBP SD were directly associated factors for poor outcomes of GMH-IVH, with an OR (95% CI) of 3.942 (1.509–6.680) and 2.334 (1.013–5.378), respectively (Table 8).

**Table 8:** Logistic regression analysis of directly correlated factors for outcome.

Variables	*β*	*SE*	*Wald χ* ^*2*^	*OR (95%CI)*	*P*
INR	3.350	1.499	4.922	3.942(1.509-6.680)	0.025
APTT	0.009	0.022	0.157	1.041(0.949-1.136)	0.692
SBP SD	0.022	0.279	0.006	1.022(0.592-1.765)	0.938
CV	0.267	0.329	0.660	1.306(0.686-2.488)	0.417
DBP Mean	−0.109	0.090	1.477	0.897(0.752-1.369)	0.224
SD	0.848	0.426	3.962	2.334(1.013-5.378)	0.047
CV	0.555	0.296	3.519	1.723(0.975-3.113)	0.061

SBP, systolic blood pressure; DBP, diastolic blood pressure; SD, standard deviation; INR, international normalized ratio; APTT, activated partial thromboplastin time; SE, Standard error; OR, Odds ratio; CI, Confdence interval.

### The predictive values of INR and DBP SD for outcome of GMH-IVH

**Fig 2:**
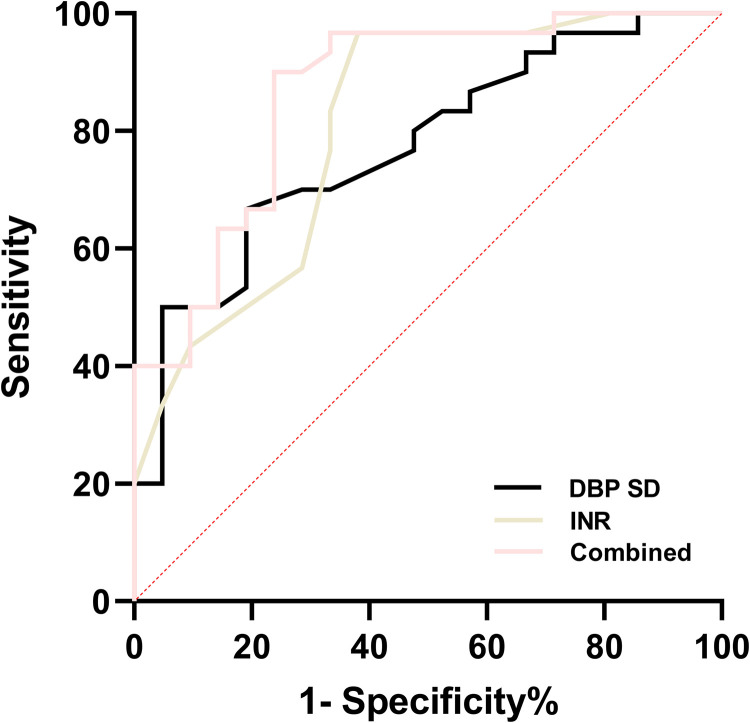
ROC curve of directly correlated factors for outcome. Combined = DBP SD + INR; DBP, diastolic blood pressure; SD, standard deviation; INR, international normalized ratio.

The combined detection of INR and DBP SD achieved the highest AUC of 0.864 for predicting poor outcomes of GMH-IVH in preterm infants, with a sensitivity of 76.2% and a specificity of 90.0% (Table 9 and Fig 2).

**Table 9:** The predictive values of INR, DBP SD, and their combination for outcome.

	AUC	95% *CI*	Cut-off	*P*	Sensitivity (%)	Specifcity (%)	Youden’s index
INR	0.808	0.683-0.933	1.85	0.001	61.9	96.7	0.586
DBP SD	0.773	0.645-0.901	6.65	<0.001	81.0	66.7	0.477
Combined	0.864	0.760-0.968	0.54	<0.001	76.2	90.0	0.662

Combined = DBP SD + INR; DBP, diastolic blood pressure; SD, standard deviation; INR, international normalized ratio; AUC, area under the curve; CI, Confdence interval.

## Discussion

GMH-IVH is significantly associated with adverse neurological outcomes, including hydrocephalus, cerebral palsy, epilepsy, and hemiplegia. In preterm infants, GMH-IVH often lacks specific clinical manifestations, and currently, there are no established methods to specifically prevent its progression [1]. In this study, we identified that elevated DBP SD and INR are correlated with the development of GMH-IVH in premature infants with a GA of ≤32 weeks. Furthermore, the combined assessment of DBP SD and INR demonstrates a certain predictive value for the early detection and prognostic evaluation of GMH-IVH.

The pathogenesis of GMH-IVH is complex and multifactorial, encompassing the following key aspects: 1) the unique vulnerability of the embryonic germinal matrix vasculature, 2) disturbances in CBF due to immature or dysfunctional cerebrovascular autoregulation (CAR), and 3) an increased risk of hemorrhage associated with abnormal coagulation function [11–13]. Furthermore, a range of perinatal factors may contribute to the development of GMH-IVH in preterm infants [14–17]. In this study, a comprehensive analysis was conducted on thirteen perinatal factors, revealing that PROM serves as a directly associated factor for GMH-IVH. Although intrauterine infection may induce PROM, the rupture of membranes also compromises the natural barrier, thereby increasing the likelihood of pathogen invasion [18]. Consequently, the presence of PROM in mothers significantly elevates the risk of perinatal infection [19]. Prior research has demonstrated that perinatal infections can trigger systemic proinflammatory cytokine responses and disrupt physiological anticoagulant and fibrinolytic mechanisms. For instance, elevated levels of inflammatory markers such as interleukin-6 have been linked to impaired coagulation function and the onset of GMH-IVH in preterm infants [18].

In prior studies, abnormal APTT and DD levels have been associated with hemorrhagic diseases in preterm infants [20]. An elevated INR within 48 hours of birth has been shown to assist in identifying preterm infants at risk for IVH [21]. Siddappa AM’s study indicated that infants with severe GMH-IVH exhibited prolonged PT and higher INR values [22]. In the current study, significant differences were observed in PT, INR, and APTT between the GMH-IVH and non-GMH-IVH groups (*P < 0.05*), with increased INR identified as a directly associated factor for GMH-IVH. These findings may be attributed to a bleeding predisposition resulting from reduced synthesis of coagulation factors or the impact of external harmful factors, which damage vascular endothelial cells and tissues, leading to the release of large quantities of tissue factors. This subsequently activates the extrinsic coagulation pathway, causing the consumption of coagulation factors. Although the role of coagulation immaturity in the pathogenesis of GMH-IVH remains controversial, the increased bleeding risk attributable to coagulation disorders is undoubtedly a contributing factor in the development of GMH-IVH [23]. This study further confirmed that there were significant differences in PT, INR, and APTT levels between the poor outcome group and the good outcome group (*P < 0.05*), with elevated INR identified as a directly associated factor for adverse outcomes in GMH-IVH. The occurrence and progression of GMH-IVH represent a dynamic evolutionary process, during which intracranial hemorrhage may also influence coagulation status, reflecting a complex interaction. Coagulation function at birth cannot adequately reflect the changing trend of coagulation function in preterm infants; therefore, dynamic monitoring of coagulation indicators is essential to further elucidate their impact on GMH-IVH.

In the early postnatal period, preterm infants are exposed to various hemodynamic instability factors, making them susceptible to hypotension, hypertension, or blood pressure fluctuations. This subsequently increases their risk of developing hyperperfusion or hypoperfusion-related brain injuries, which may be associated with the onset and progression of GMH-IVH [24–26]. BPV can mitigate the impact of pathological or physiological factors and more accurately reflect systemic blood pressure fluctuations. Our previous research has demonstrated that elevated BPV may serve as an early indicator of CAR dysfunction, indirectly reflecting fluctuations in CBF to a certain extent [27]. CAR is a pressure regulation system. When the fluctuation of cerebral perfusion pressure exceeds the range of CAR, pressure-passive CBF occurs, which may lead to intracranial hemorrhage [24,25]. In this study, we identified a significant association between BPV and GMH-IVH (*P < 0.05*), with increased DBP SD serving as a directly correlated factor for GMH-IVH. Consequently, higher BPV correlates with an elevated development of GMH-IVH. However, there is currently no reported evidence regarding whether BPV can serve as an indicator for evaluating the prognosis of GMH-IVH. This study demonstrated that increased BPV was associated with short-term outcomes in preterm infants with GMH-IVH, and elevated DBP SD acts as a directly correlated factor for adverse short-term outcomes. Therefore, early implementation of blood pressure monitoring and management, along with minimizing blood pressure fluctuations, could be beneficial in preventing the occurrence of GMH-IVH and mitigating its adverse effects in preterm infants.

Currently, despite the availability of alternative methods, ultrasound remains the primary modality for detecting GMH-IVH. However, the diagnosis may be easily overlooked due to factors such as limited visibility or concealed locations, especially in the early stages [28]. The Papile classification has been utilized as a primary screening method for GMH-IVH among preterm infants in our center. Its advantages include simplicity and ease of application, enabling clinicians to acquire proficiency within a relatively short timeframe. These characteristics render it particularly suitable for initial large-scale and rapid screening procedures. Furthermore, implementing ultrasound monitoring specifically for high-risk populations not only facilitates the early detection of GMH-IVH, but also avoids the substantial resource consumption associated with comprehensive continuous screening. In this study, BPV and coagulation indices, which are commonly utilized in clinical practice due to their ease of acquisition and monitoring, were employed to predict the risk population for GMH-IVH. The results demonstrated that the combined detection of INR and DBP SD exhibited a certain predictive value for GMH-IVH occurrence in preterm infants, with a sensitivity of 82.4% and specificity of 79.7%. Therefore, it is imperative to give adequate attention to preterm infants exhibiting significantly abnormal coagulation function and high BP fluctuations in clinical settings, enhance monitoring protocols, and implement comprehensive management strategies aimed at reducing BP fluctuations and improving coagulation function, thereby mitigating the risk of GMH-IVH.

GMH-IVH frequently presents without symptoms or with only non-specific symptoms, leading to a higher likelihood of being overlooked in clinical diagnosis. Currently, there is no established effective treatment for GMH-IVH, which significantly increases the risk of poor prognosis. Severe GMH-IVH is strongly associated with neurodevelopmental impairments and elevated mortality rates [29]. However, the impact of mild GMH-IVH on neurodevelopment remains a subject of debate [30]. A prior study demonstrated that patients with isolated grade II GMH-IVH exhibited learning difficulties as well as cognitive and executive functional deficits [31]. Consequently, it is imperative to focus on the early prediction of neurodevelopmental adverse outcomes in preterm infants with GMH-IVH. Our findings revealed that abnormal INR and DBP SD were independent influencing factors for the outcome of GMH-IVH in preterm infants with GA ≤ 32 weeks. The combined assessment of INR and DBP SD achieved the highest AUC of 0.864, with a sensitivity of 76.2% and specificity of 90.0%. Therefore, we speculate that in clinical practice, close monitoring is essential for premature infants with GMH-IVH who exhibit significantly prolonged INR, increased BPV, and an absence of overt symptoms, and that reference to cranial MRI examinations may be beneficial in assessing the risk of poor prognosis. Timely neurological intervention and focus on follow-up should be carried out for GMH-IVH infants with increased risk of poor short-term outcome.

## Limitation

This study has several limitations. First, it was conducted as a single-center study with a relatively limited sample size. Second, there was an absence of a fully unified and precise standard for the reference range of coagulation function in preterm infants across different gestational ages. Additionally, owing to the restricted sample size, we were unable to conduct a detailed analysis of the relevant reference ranges. Third, the duration of follow-up was relatively brief. Consequently, future prospective multicenter studies with larger sample sizes and extended follow-up periods are necessary to comprehensively evaluate the impact of GMH-IVH on nervous system development.

## Conclusions

Currently, there is no universal consensus regarding the definition of normal BP in preterm infants, and utilizing the optimal BP range as the criterion for evaluating CBF stability remains controversial. Predicting the risk of GMH-IVH in preterm infants based solely on hypotension or hypertension lacks both accuracy and comprehensiveness. Our study demonstrated that increased INR and DBP SD are directly correlated factors for the development and poor short-term outcomes of GMH-IVH. Moreover, combined monitoring of INR and DBP SD holds certain reference value for the early identification and prognostic evaluation of GMH-IVH in preterm infants with a GA of ≤32 weeks. Reducing blood pressure fluctuations and correcting coagulation dysfunction in the early postnatal period may contribute to the prevention and improvement of GMH-IVH outcomes in preterm infants.

## Supporting information

S1 Fig Study flow chart. (TIF)
