# Supplementary figures and images for: Blood pressure variability combined with coagulation function in early prediction and outcome assessment of germinal matrix-intraventricular hemorrhage in preterm infants with gestational age ≤32 weeks
Source: PLoS One. 2025 Jul 24;20(7):e0328904. doi: 10.1371/journal.pone.0328904 (PMC12289036; doi:10.1371/journal.pone.0328904)

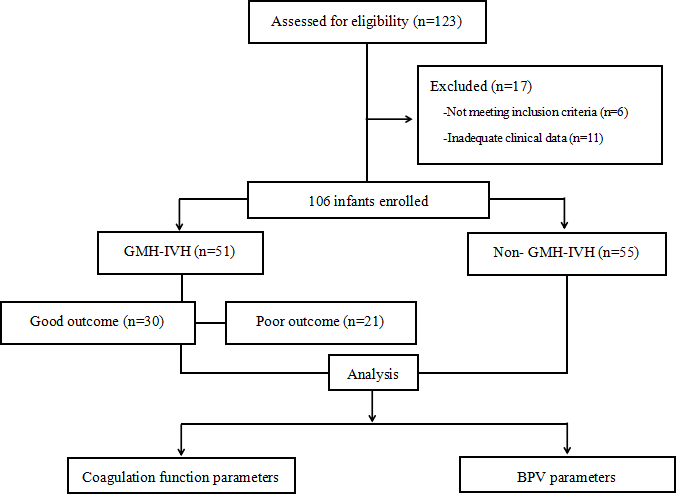

Supplement: S1 Fig — (TIF) [file pone.0328904.s001.tif]
